# Biology of platelet-rich plasma and its clinical application in cartilage repair

**DOI:** 10.1186/ar4493

**Published:** 2014-02-25

**Authors:** Xuetao Xie, Changqing Zhang, Rocky S Tuan

**Affiliations:** 1 Department of Orthopaedic Surgery, Shanghai Sixth People's Hospital, Shanghai Jiaotong University School of Medicine, Shanghai 200233, China; 2 Center for Cellular and Molecular Engineering, Department of Orthopaedic Surgery, University of Pittsburgh School of Medicine, Pittsburgh, PA 15219, USA

## Abstract

Platelet-rich plasma (PRP) is an autologous concentrated cocktail of growth factors and inflammatory mediators, and has been considered to be potentially effective for cartilage repair. In addition, the fibrinogen in PRP may be activated to form a fibrin matrix to fill cartilage lesions, fulfilling the initial requirements of physiological wound healing. The anabolic, anti-inflammatory and scaffolding effects of PRP based on laboratory investigations, animal studies, and clinical trials are reviewed here. *In vitro*, PRP is found to stimulate cell proliferation and cartilaginous matrix production by chondrocytes and adult mesenchymal stem cells (MSCs), enhance matrix secretion by synoviocytes, mitigate IL-1β-induced inflammation, and provide a favorable substrate for MSCs. In preclinical studies, PRP has been used either as a gel to fill cartilage defects with variable results, or to slow the progression of arthritis in animal models with positive outcomes. Findings from current clinical trials suggest that PRP may have the potential to fill cartilage defects to enhance cartilage repair, attenuate symptoms of osteoarthritis and improve joint function, with an acceptable safety profile. Although current evidence appears to favor PRP over hyaluronan for the treatment of osteoarthritis, the efficacy of PRP therapy remains unpredictable owing to the highly heterogeneous nature of reported studies and the variable composition of the PRP preparations. Future studies are critical to elucidate the functional activity of individual PRP components in modulating specific pathogenic mechanisms.

## Introduction

Cartilage injuries are a common clinical challenge and affect 27 million people in the United States, resulting in 208,600 primary total hip replacement and 450,000 primary total knee replacements, according to data for 2005 [1,2]. The number of total hip replacement and total knee replacement operations is expected to reach 572,000 and 3,480,000, respectively, by 2030 [1].

**Figure 1:**
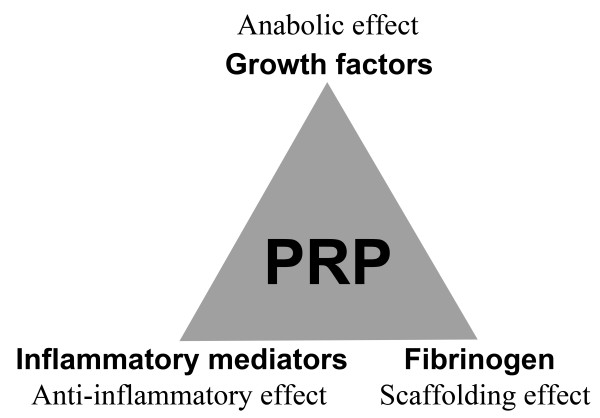
**Principal components and potential effects and actions of PRP.** PRP contains growth factors that stimulate cellular anabolism, inflammatory mediators and modulators that exert anti-inflammatory effects, and fibrinogen that acts as a biomaterial scaffold. PRP, platelet-rich plasma.

In the past decade, platelet-rich plasma (PRP) has emerged as a non-operative treatment modality for cartilage injuries [3,4]. The rationale for its use is largely dependent on its functional components (Figure 1).While there are significant variations in its makeup, the initial PRP consistently contains highly concentrated platelets and a number of plasma proteins associated with platelets during its preparation by centrifugation.

Platelets are produced by megakaryocytes as anucleated cells [5]. A variety of growth factors, coagulation factors, adhesion molecules, cytokines, chemokines and integrins are stored in platelets [6-8]. After activation, the platelets in PRP can release a multitude of growth factors at concentrations significantly higher than the baseline blood levels, including transforming growth factor-β, platelet-derived growth factor (PDGF), insulin-like growth factor (IGF), basic fibroblast growth factors, vascular endothelial growth factor (VEGF), epidermal growth factors, and many others [9]. Many of these anabolic cytokines, such as transforming growth factor-β, IGF, basic fibroblast growth factor and PDGF, are chondro-promoting and chondro-protective [10-13]. Specifically, they can stimulate chondrocyte and multipotent mesenchymal stem cell (MSC) proliferation, promote chondrocyte synthesis of aggrecan and collagen type II (Col II), drive MSC chondrogenic differentiation, prevent chondrocyte and MSC apoptosis, and diminish the catabolic effects of inflammatory cytokines, such as IL-1β, and matrix metalloproteinases (MMPs).

Platelets in PRP are also a source of inflammatory mediators and modulators. After incubation with polyacrylamide beads, platelets may release numerous anti-inflammatory cytokines, including IL-1 receptor antagonist (IL-1ra), soluble tumor necrosis factor (TNF) receptor (sTNF-R) I and II, IL-4, IL-10, IL-13, and interferon γ [14]. Specifically, IL-1ra inhibits the bioactivity of IL-1 by blocking its receptors [15,16]. sTNF-RI and sTNF-RII can bind to free TNFα, thereby preventing signal transduction [15,16]. IL-4, IL-10 and IL-13 can increase IL-1ra production and reduce TNFα-induced prostaglandin E2 production [17,18]. Interferon γ induces the production of IL-18-binding protein, a natural inhibitor of IL-18 [19]. Although PRP also releases pro-inflammatory cytokines, such as IL-1α, IL-1β, TNFα, IL-6, IL-8, IL-17 and IL-18, their concentrations are much lower than those of the anti-inflammatory counterparts [14]. For instance, the concentration of IL-1ra is over 23,000 times higher than that of IL-1α and over 8,000 times than that of IL-1β in PRP. The significant difference between the concentrations of anti-inflammatory cytokines and those of the pro-inflammatory factors in PRP suggests that PRP may suppress inflammation in osteoarthritis (OA), thereby protecting cartilage and reducing pain.

PRP also contains a variety of plasma proteins, which are known to be critical components in the healing mechanism of connective tissues [20]. Different from serum, plasma contains fibrinogen and other clotting factors, which can be activated to form a provisional fibrin scaffold for cells to adhere, migrate and proliferate [21]. Since platelets aggregate along the fibrin fibers during clotting, the resultant three-dimensional scaffold can also act as a reservoir of growth factors that exert favorable effects on cells [21,22]. The clinical benefits of the PRP fibrin matrix have been well-known in maxillofacial surgery and chronic wound repair [23,24]. As articular cartilage contains no blood vessels and is thus unable to initiate the same healing process as other tissues with good regenerative potential, the introduction of the PRP scaffold may mimic the initial stage of wound healing and tissue repair.

Based on the potential benefits of its component biological factors, it has been hypothesized that PRP or its derivatives may have positive effects on cartilage repair. Since there are extensive reviews on specific growth factors in the literature [25-27], this review will focus mainly on the collective effects of PRP on cells, including chondrocytes, MSCs from various tissue origins, and synoviocytes, and on cartilage injury in laboratory animal models, including equines, and human cartilage diseases. In order to provide an accurate overview, the classification system advocated by Dohan and colleagues [28] is adopted here to categorize generic PRP into pure PRP (P-PRP), leukocyte- and PRP (L-PRP), pure platelet-rich fibrin, and leukocyte- and PRF (L-PRF), whenever relevant information is available.

## Effect of platelet-rich plasma on cells

### Chondrocytes

#### Proliferation

In almost all published studies, PRP has been shown to have a strong positive effect on chondrocyte proliferation *in vitro* (Table 1) [29-34]. When adult porcine chondrocytes were cultured in alginate beads in the presence of 10% PRP releasate (PRPr), 10% platelet-poor plasma (PPP) releasate or 10% fetal bovine serum (FBS) for 72 hours, the increase in DNA content in the PRP group was significantly higher than that in the PPP group and that in the FBS group [29]. The stimulatory effect of PRP on chondrocyte proliferation was also observed after 4, 8 and 12 days of *in vitro* culture, in either a monolayer or three-dimensional environment [30]. Additional studies revealed a dose- and time-dependent enhancement of chondrocyte proliferation by PRPr, even during the 20 day monolayer culture [31,32].

**Table 1:** **Summary of *in vitro *** **effects of platelet-rich plasma on chondrocytes**

**Cell type**	**Intervention**	**Outcome**	**Reference**
Porcine chondrocytes	10% PRP releasate after thrombin and CaCl_2_ activation	Increased cell proliferation, proteoglycan and Col II synthesis	[29]
Human osteoarthritic chondrocytes	Bovine fibrin + L-PRF releasate on two-dimensional surface and in three-dimensional scaffold	Increased cell proliferation and Col II and aggrecan mRNA expression and GAG and proteoglycan accumulation	[30]
Human osteoarthritic chondrocytes	5% PRP releasate obtained by two cycles of freezing and thawing	Increased cell proliferation, proteoglycan synthesis, Sox-9 and aggrecan mRNA expression and proteins associated with chondrocyte differentiation	[31]
Bovine chondrocytes	Platelet supernatant	Stimulated proliferation, but failed to induce deposition of typical cartilaginous ECM	[32]
Human chondrocytes	1% or 10% platelet supernatant (leukocyte-filtered)	Accelerated cell expansion, but reduced Col II mRNA expression and induced chondrocytes towards a fibroblast-like phenotype	[33]
Sheep chondrocytes	Double-spun PRP activated by CaCl_2_	Stimulated cell proliferation, but reduced Col II mRNA expression	[34]
Rabbit chondrocytes	Hydrogel + chondrocytes with double-spun PRP	Enhanced chondrogenic differentiation and maturation with up-regulation of CB1 and CB2	[36,37]
Human osteoarthritis chondrocytes	Gelatin microcarriers + biological glues (whole blood, PPP, PRP, or commercial fibrin glue)	No difference in ECM production between any two of these groups	[38]
Human osteoarthritic chondrocytes	10% L-PRP releasate after CaCl_2_ activation	Decreased IL-1β-induced inflammatory effects and inhibited NF-κB activation	[39]
Immortalized human chondrocytes	PRP releasate activated by CaCl_2_	Decreased COX-2 expression and inhibited NF-κB activation via HGF and TNF-α	[40]

Col, collagen; ECM, extracellular matrix; GAG, glycosaminoglycan; HGF, hepatocyte growth factor; L-PRF, leukocyte- and platelet-rich fibrin; L-PRP, leukocyte- and platelet-rich plasma; NF-κB, nuclear factor kappa B; PPP, platelet-poor plasma; PRP, platelet-rich plasma.

#### Differentiation

There is, however, less concordance in the effect of PRP on chondrocyte differentiation. Akeda and colleagues [29] reported that 10% PRP treatment significantly increased proteoglycan and Col II synthesis compared to treatments with 10% PPP or 10% FBS, with the major profiles of proteoglycan and collagens being similar to those seen in cells cultured with FBS, indicating maintenance of a stable chondrocyte phenotype with PRP exposure. Similar effects were also noted in human osteoarthritic chondrocytes [30]. The supernatant from platelet-rich fibrin (PRF) up-regulated the mRNA expression of Col II and aggrecan and increased synthesis of glycosaminoglycan and proteoglycan by chondrocytes cultured either on the two-dimensional surface of fibrin scaffolds or in three-dimensional scaffolds compared to controls without addition of exudate. Furthermore, a proteomic study revealed that PRPr supplementation could also induce the expression of proteins associated with chondrocyte differentiation [31]. In particular, PRPr increased the expression of aggrecan and Sox9, without increasing the expression of Col X and alkaline phosphatase. Increased Sox9 expression has been shown to be associated with the chondrocytic re-differentiation process [35]. These results show that PRP had differential effects on chondrocytes; that is, promoting the synthesis of hyaline cartilage matrix while cellular progression to terminal hypertrophy is not facilitated or at least delayed. Another study further demonstrated that the chondrogenic differentiation and maturation induced by PRP treatment was related to the up-regulated expression of cannabinoid receptor 1 and 2 [36,37]. However, a few authors have argued that PRP treatment was unable to induce the deposition of typical cartilage matrix components [32,34], that there was no difference in the enhancement of extracellular matrix (ECM) production between the groups with PRP, PPP, whole blood or fibrin glue added into gelatin-based microcarriers [38], and that PRP treatment could in fact induce a dedifferentiation of chondrocytes towards a fibroblast-like phenotype [33]. This lack of consistency among the published reports may be attributable to the heterogeneity of study designs, variations in PRP preparations, and differences in PRP delivery. For instance, some studies used platelet lysate through repeated freeze-thawing after centrifugation, some employed the exudate after clot formation without addition of external activators, while others adopted PRPr collected after thrombin activation [29-31].

#### Anti-inflammation

PRP has also been demonstrated recently to have anti-inflammation potential in an osteoarthritic milieu. Human osteoarthritic chondrocytes were cultured with 10 ng/ml IL-1β to mimic an osteoarthritic environment [39] in medium with or without 10% PRPr. After 48 hours, IL-1β inhibited Col II and aggrecan gene expression and concomitantly increased expression of a disintegrin and metalloproteinase with thrombospondin motifs-4 and prostaglandin-endoperoxide synthase-2, whereas PRPr supplementation reduced these IL-1β-mediated effects. In addition, the IL-1β-induced activation of nuclear factor kappa B (NF-κB), a major pathway involved in the pathogenesis of OA, could be completely inhibited by PRP (*P* < 0.001). Further study revealed that PRPr inhibited NF-κB activation through increasing gene expression of hepatocyte growth factor (HGF) and TNF-α [40]. HGF has been shown to increase NF-κB inhibitor-α expression, thus impairing p65 translocation to the nucleus, which is necessary for NF-κB activation [41,42]. TNF-α enhances p50 homodimer formation and its binding to DNA to inhibit NF-κB pathway activation [43]. It is noteworthy that the NF-κB pathway is not the only one involved in the PRP anti-inflammatory activities. IGF-1 and PDGF-bb present in PRP could also suppress the activation of the Src/PI3K/AKT pathway, thus inhibiting chondrocyte apoptosis and inflammation induced by IL-1β [44].

It should also be noted that some PRP formulations could be pro-inflammatory [45]. The presence of concentrated leukocytes increased the levels of catabolic and pro-inflammatory signaling molecules, including MMPs and IL-1β [46]. In addition, activated platelets could produce IL-1β to mediate pro-inflammatory signaling [47]. However, the most represented pro-inflammatory cytokine, IL-1β, only showed a slight increase after platelet activation, whereas the anti-inflammatory molecules, such as IL-4 and IL-10, increased more than five times [40]. A recent study confirmed the dual effect of platelet lysate on human chondrocytes - a transient pro-inflammatory activity followed by an inflammation resolution [48]. Although the net results of PRP are variable owing to compositional heterogeneity, the anti-inflammatory effect is likely to predominate in PRP formulations in which the presence of leukocytes is substantially reduced.

### Adult mesenchymal stem cells

As candidate cells applicable for tissue engineering-based approaches to cartilage repair, MSCs have noticeable advantages over chondrocytes due to their abundant availability, robust chondrogenic activity accompanied by cartilage matrix production, and multi-lineage differentiation ability to repair osteochondral defects [49-51].

#### Bone marrow-derived mesenchymal stem cells

Among their various tissue sources, MSCs derived from bone marrow (BMSCs) are employed most extensively in cartilage engineering. Many researchers have found that PRP exhibits a mitogenic effect on MSCs (Table 2) [34,52-55]. When human BMSCs in monolayer were cultured with 10% inactivated autologous PRP, a five-fold increase in cellular proliferation was seen at day 7 relative to the control without PRP supplementation [56]. While both chondrogenic and osteogenic gene markers were up-regulated in the presence of PRP, the chondrogenic markers, including Sox9 and aggrecan, increased much more (over 10-fold increase) than RUNX2 (less than 2-fold increase) [56], a marker of early osteogenic differentiation [57]. The authors concluded that PRP could enhance the proliferation and chondrogenic differentiation of BMSCs. In this study, however, BMSCs were cultured for only 7 days, and the long-term effects of PRP on MSCs thus remained unclear. In another study with 21 days of BMSC culture, expression of Sox9, aggrecan and Col II increased significantly at both the mRNA and protein levels in the presence of inactivated PRP, compared to the FBS control [21]. However, Col I gene and protein expression was also increased concomitantly by PRP. This non-selective up-regulation of both chondrogenic and osteogenic genes by PRP in multipotent BMSCs *in vitro* must be considered with caution in cartilage tissue engineering, but may be beneficial and applicable for repairing osteochondral defects.

**Table 2:** **Summary of effects of platelet-rich plasma on mesenchymal stem cells from various tissue sources *in vitro***

**Cell type**	**Intervention**	**Outcome**	**Reference**
Sheep BMSCs	Double-spun PRP activated by CaCl_2_	Increased cell proliferation and Col II mRNA expression	[34]
Human BMSCs	50% platelet lysate after two cycles of freezing and thawing	Promoted proliferation and triggered chondrogenic differentiation	[55]
Human BMSCs	10% inactivated PRP (leukocyte concentration unreported)	Enhanced cell proliferation and Sox9, aggrecan and RUNX2 mRNA expression	[56]
Rabbit BMSCs, ADSCs	10% double-spun inactivated PRP	Increased cell proliferation and expression of Sox9, aggrecan, Col II and Col I mRNA and proteins	[21]
Mouse MDSCs	Double-spun PRP	Promoted cell proliferation, adhesion and migration of MDSCs, and increased number of cells producing Col II and cell apoptosis	[58]
Human subchondral progenitor cells	5% P-PRP after freezing and thawing	Increased cell migration and cartilaginous matrix formation, but did not affect osteogenic and adipogenic differentiation	[60]

ADSC, adipose-derived mesenchymal stem cell; BMSC, bone marrow-derived mesenchymal stem cell; Col, collagen; MDSC, muscle-derived mesenchymal stem cell; P-PRP, pure platelet-rich plasma; PRP, platelet-rich plasma.

#### Adipose- and muscle-derived stem cells and human subchondral bone-derived progenitor cells

Besides BMSCs, the effect of PRP on MSCs derived from fat, muscle and subchondral bone has also been preliminarily studied. Rabbit adipose-derived MSCs responded to PRP stimulation in a manner similar to BMSCs, in that cell proliferation, gene and protein expression of Sox9, aggrecan, Col I and Col II were enhanced significantly compared to the FBS controls [21]. When muscle-derived MSCs were cultured in the presence of PRP, their ability to proliferate, adhere and migrate was significantly promoted [58]. Although chondrogenic gene expression was not up-regulated, the number of cells producing Col II was increased markedly. Meanwhile, cellular apoptosis was also increased *in vitro*, but *in vivo* study yielded contrary results showing that apoptosis was suppressed in the presence of PRP [58]. MSCs from the subchondral bone are considered the main cell sources responsible for the repair of cartilage defects in the clinical procedure of microfracture [59]. A study reported that P-PRP treatment could stimulate the vertical migration of subchondral progenitors and cartilaginous matrix accumulation, including proteoglycan and Col II [60]. More importantly, while chondrogenic differentiation of the progenitor cells was induced significantly by PRP treatment, osteogenic and adipogenic differentiation were not affected. These findings suggest that PRP might accelerate the migration of the subchondral progenitors to repair cartilage defects with the formation of hyaline cartilage.

#### Scaffolding activity

In addition to the positive effects on MSC proliferation, differentiation and migration, PRP may also provide a three-dimensional substrate for cell seeding by virtue of the presence of fibrinogen, which gives rise readily to fibrin gel upon thrombin or calcium activation. In a recent study, about 1 × 10^5^ rabbit BMSCs were mixed with 60 μl ultra-filtered platelet lysate and the composite was then activated by thrombin and CaCl_2_ to form a three-dimensional cell-laden scaffold, followed by *in vitro* culturing in chondrogenic induction medium for 21 days [61]. At 1 week, round-shaped chondrocyte-like cells were found homogeneously distributed inside lacunae and some cells clustered together within the scaffold. Histological analysis at 3 weeks further confirmed the presence of these chondrocyte-like cells and the accumulation of cartilaginous ECM deposition. However, the details of the structure of this scaffold were not investigated.

In another study, Kang and colleagues [22] examined the components and microstructure of L-PRF, which formed naturally during the single step centrifugation of whole blood without anti-coagulants. They found that there were two distinct zones in the PRF scaffold, the platelet zone and the fibrin zone. The marked advantage of L-PRF is the ease of the procedure and the absence of additional chemicals. Nonetheless, the PRF preparation may not allow cells to seed evenly inside, and its two-zone microstructure implies possible large variations in the release of growth factors.

**Figure 2:**
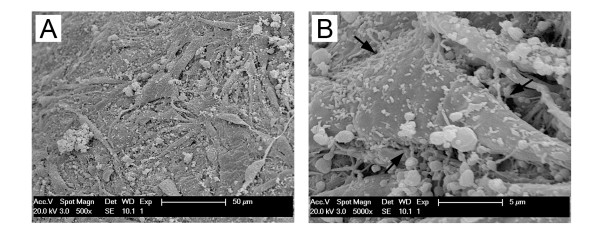
**Scanning electron microscopy of MSC-laden PRP scaffolds.** MSCs adhere to the PRP fibrin fibers (adhesion sites indicated by arrows in B). **A)** low magnification; **B)** high magnification. MSC, mesenchymal stem cell; PRP, platelet-rich plasma.

In our recent study, we adopted the traditional double spinning method to prepare liquid PRP first. Then MSCs were distributed in the PRP before it was activated by CaCl_2_ to form a three-dimensional scaffold. Histological and scanning electron microscopy evaluations revealed that the PRP matrix had a honeycomb microstructure with platelets aggregated along the fibrin skeleton to which MSCs adhered (Figure 2) [21]. Another pilot study demonstrated that equine BMSCs cultured in the three-dimensional PRP gel for 3 weeks resulted in enhanced cell proliferation and proteoglycan synthesis compared to those in the fibrin gel alone [3]. Unlike L-PRF, however, both the platelet lysate and the double-spun PRP activated by CaCl_2_ usually have a low density of fibrin and weak polymerization, and thus dissolve quickly, similar to fibrin glue.

It was reported that commercially available fibrin gel would disintegrate within 7 days, but such a rapid degradation could be delayed by seeding cells and adding fibrinolytic inhibitors, such as tranexamic acid, aprotonin, and galardin [62-64]. PRP-tranexamic acid gel seeded with chondrocytes could maintain stability *in vitro* for 4 weeks without any shrinkage, while cells remained viable and were able to migrate [64]. The seeded chondrocytes likely produced abundant ECM, in a manner commensurate with scaffold degradation [62]. Inhibition of fibrin degradation would also mean slower and more extended release of growth factors to produce better reparative results [65]. The low mechanical property of the PRP fibrin scaffold may be improved through genipin cross-linking [66], ruthenium-catalyzed photo cross-linking [67], or adjusting the content of fibrinogen [68]. An optimized fibrin gel could resist dynamic compression and shear at the tissue site, while the embedded chondrocytes continued to produce a coherent cartilaginous ECM, containing proteoglycan and Col II [66,68]. In addition, PRP gel may also be employed with other biomaterials to enhance its mechanical properties.

### Synoviocytes

Intra-articular use of PRP may also have an effect on fibroblast-like synoviocytes, which can secrete hyaluronic acid (HA) and HGF and produce cytokines and MMPs found in synovial fluid [69]. HA in the synovial fluid has been shown to have beneficial effects for arthritic patients [70], while HGF is involved in many signaling pathways and has been shown to inhibit NF-κB activation [40]. On the other hand, MMPs can mediate cartilage catabolism [71]. The effect of PRP on synoviocytes may thus indirectly affect the repair of cartilage injury. Synoviocytes from OA patients cultured in 20% single-spun P-PRP activated by CaCl_2_ produced significantly more HA and HGF compared to those cultured in PPP [72]. P-PRP-enhanced HA secretion was also observed in synoviocytes in the presence of IL-1β, indicating that PRP could enhance chondroprotection and joint lubrication via synoviocytes even in the face of inflammation. L-PRP-treated human synoviocytes exhibited significantly higher levels of MMPs than untreated synoviocytes [73], but P-PRP did not exacerbate the IL-1β-induced rise of MMPs in synoviocytes from OA patients [72].

## Effect of platelet-rich plasma on cartilage repair in animal models *in vivo*

### Cartilage defect models

In order to investigate whether PRP has a positive effect on chondrogenesis *in vivo*, Wu and colleagues [74] distributed cultured chondrocytes within autologous PRP to generate PRP/chondrocyte composites with a final cell density of 5 × 10^7^/ml. The composites were activated by thrombin before they were injected into the dorsal subcutaneous tissue of the donor rabbits. After 2 months, hard palpable nodules were formed under the skin and magnetic resonance imaging (MRI) evaluation demonstrated the presence of cartilage-like tissue. Safranin-O staining and Masson's trichrome histological staining indicated the presence of proteoglycan and collagen. Although the type of the newly regenerated collagen was not determined, these findings suggested that PRP could be used as a cell scaffold in cartilage tissue engineering (Table 3). In another study, PRP was used as a bioactive scaffold alone or with MSCs to fill osteochondral defects (4 mm in diameter, 3 mm in depth) in the femoropatellar groove of rabbits [21]. At 12 weeks post-implantation, PRP scaffold alone yielded better macroscopic, histological and immunological results than those in the untreated group, but worse than those of the PRP-MSC group. Both immunohistological and molecular evaluation demonstrated the presence of Col II in the regenerated tissue in the PRP scaffold group and more abundant Col II accumulation in the PRP-MSC group. In light of the poor mechanical properties of PRP, Sun and colleagues [75] used PRP as an additive to poly (lactic-co-glycolic acid) (PLGA) scaffolds to repair large osteochondral defects (5 mm in diameter, 4 mm in depth) in rabbits. After 4 and 12 weeks, compared to PLGA alone, PRP increased the content of cartilaginous ECM, and improved subchondral bone formation. These results were consistent with those from a previous study that used bilayer collagen scaffold with or without PRP to repair osteochondral defects (4 mm in diameter, 3 mm in depth) in rabbits [76].

**Table 3:** Summary of animal studies of platelet-rich plasma for treatment of cartilage defects

**Animal model**	**Defect size**	**Intervention**	**Outcome**	**Reference**
Rabbit osteochondral defect in trochlea	4 mm diameter, 3 mm depth	Untreated; double-spun PRP activated by CaCl_2_; PRP gel + ADSCs; PRP gel + BMSCs	PRP group yielded better macroscopic and histological results than untreated, but worse than PRP with cells	[21]
Rabbit osteochondral defect in trochlea	5 mm diameter, 4 mm depth	Untreated; double-spun PRP activated by thrombin and CaCl_2_ + PLGA; PLGA	Macroscopic examination, micro-CT, and histology of newly formed osteochondral tissue differed significantly between PRP-treated and untreated groups	[75]
Rabbit osteochondral defect in trochlea	4 mm diameter, 3 mm depth	Untreated; collagen scaffold alone or with doule-spun inactivated PRP	PRP-collagen group had highest histological scores and most GAG content; mechanical property was only better than that in the untreated group.	[76]
Sheep osteochondral defect in femoral condyle	7 mm diameter, 9 mm depth	Untreated; collagen-hydroxyapatite scaffold alone or with L-PRP activated by CaCl_2_	Good integration of the chondral surface in both treatment groups; better osteochondral reconstruction in the group treated with scaffold alone than with PRP	[77]
Goat osteochondral defect in trochlea	6 mm diameter, 0.8 mm depth	Engineered cartilage implants with periosteal flap or L-PRP or human fibrin	PRP and human fibrin glue interfered with retention of the implants and integration with adjacent cartilage	[78]
Sheep chondral defect in femoral condyle	8 mm diameter, cartilage only	Microfracture alone or with five weekly P-PRP intra-articular injections	PRP enhanced the macroscopic, histological and biomechanical characteristics at 3 months, 6 months and 12 months, but did not produce hyaline cartilage	[79]
Sheep chondral defect in femoral condyle	8 mm diameter, cartilage only	Microfracture alone, with single P-PRP injection or with P-PRP and fibrin gel filling up the defects	PRP with fibrin gel yielded the best histological results and biomechanical results, close to those of the normal cartilage, but still did not produce hyaline cartilage	[80]

ADSC, adipose-derived mesenchymal stem cell; BMSC, bone marrow-derived mesenchymal stem cell; CT, computed tomography; GAG, glycosaminoglycan; L-PRP, leukocyte- and platelet-rich plasma; PLGA, poly (lactic-co-glycolic acid); P-PRP, pure platelet-rich plasma; PRP, platelet-rich plasma.

However, the beneficial effect of PRP on cartilage regeneration in rabbits could not be consistently verified in larger animal models, including sheep and goats. In a sheep model, osteochondral defects (7 mm in diameter, 9 mm in depth) were created in the femoral condyles and then filled with collagen-hydroxyapatite scaffold alone or in combination with L-PRP [77]. Although good integration of the chondral surface was achieved in both treatment groups at 6 months, incomplete bone formation and irregular cartilage surface integration were observed in the PRP-treated group, whereas significantly better osteochondral reconstruction was seen in the group treated with the scaffold alone. Another study using a goat model of shallow osteochondral defects (6 mm in diameter, 0.8 mm in depth) also confirmed the inhibitory effect of L-PRP addition on cartilage repair [78]. Nonetheless, according to the study by Milano and colleagues [79], repeated P-PRP intra-articular injections in a sheep chondral model (8 mm in diameter, cartilage only) significantly improved the macroscopic, histological and biomechanical outcomes of the newly regenerated tissue compared to the group without PRP injections. In addition, the reparative response after P-PRP injections was more durable and stable during the 12-month observation period. Their later study showed that stabilizing P-PRP in the chondral defects with fibrin glue resulted in higher scores in histological and mechanical assessment than PRP intra-articular injections, but neither technique resulted in the regeneration of hyaline cartilage [80]. These conflicting results suggest the negative effect of concentrated leukocytes in PRP formulations on cartilage repair.

### Arthritis models

In a traumatic OA model in rabbits induced by unilateral transection of the anterior cruciate ligament, 33 rabbits were treated with phosphate buffered saline (PBS) injections, PBS-microsphere injections, injections of PRPr after thrombin and CaCl_2_ activation, or injections of microspheres containing PRPr into the affected knee joints; injections were given twice with a 3-week interval between them (Table 4) [65]. Ten weeks later, severe OA changes (erosion to subchondral bone) were observed in 25% of the PBS group, 33% of the PBS-microsphere group and 25% of the PRPr group, but none of the joints in the PRPr-microsphere groups showed severe OA. This finding suggested that the sustained release of PRPr had an inhibitory effect on the progression of traumatic OA. The effect of PRP on existing advanced OA remains to be investigated. In a non-traumatic OA model in rabbits induced by twice intra-articular injections of collagenase type II, single-spun PRP was injected 4 weeks later, with saline infiltration as a control [81]. Four weeks later, the PRP-injected knees showed significantly reduced macroscopic and histologic cartilage degeneration scores compared to the control, suggesting that PRP could suppress the catabolic effect of collagenase or enhance the anabolic response of the damaged cartilage. However, neither of the above studies investigated changes in the inflammatory mediators involved. PRP has also been tested for the treatment of rheumatoid arthritis of joints. In a pig model, bovine serum albumin was administered to induce arthritis in bilateral knees [82]. Autologous PRP was injected into the right knees 2 weeks and 4 weeks later, with saline injections into the left knees as the control group. At 2 weeks after the second injection, PRP treatment significantly attenuated the decrease in proteoglycan and Col II contents in cartilage, the increase in IL-6 and VEGF levels, and the elevation in protein concentrations of IL-1β, IL-6, VEGF, and IGF-1 in synovium and cartilage, compared to the control. These results not only showed that PRP might be an alternative treatment for acute rheumatoid arthritis, but also supported the notion that PRP had anti-inflammatory effects on joint arthritis.

**Table 4:** Summary of animal studies of platelet-rich plasma for treatment of knee arthritis

**Model or disease**	**Intervention**	**Outcome**	**Reference**
Traumatic OA model in rabbits induced by ACLT	Injections of PBS, PBS-microspheres, PRPr after thrombin and CaCl_2_ activation or PRPr-microspheres	OA occurred in 25% of the PBS group, 33% of the PBS-microsphere group, and 25% of the PRP group, but no joints in the PRP-microsphere group showed OA changes at 10 weeks	[65]
Non-traumatic OA model in rabbits induced by collagenase	P-PRP or saline intra-articular injection at 4 weeks after collagenase infiltration	Significantly lower macroscopic and microscopic scores in the PRP-treated group than in the saline-treated group at 8 weeks	[81]
BSA-induced rheumatoid arthritis model in pigs	Double-spun, inactivated PRP intra-articular injections or saline at 2 weeks and 4 weeks after BSA injection	PRP suppressed the decrease of proteoglycan and Col II content in cartilage and the increase of inflammatory cytokines in synovium and cartilage induced by BSA at 6 weeks	[82]
Primary OA or osteochondrosis in horses	Three P-PRP intra-articular injections at 2 week intervals	PRP diminished synovial effusion and lameness in the affected joints significantly during 1 year follow-up	[83]

ACLT, anterior cruciate ligament transection; BSA, bovine serum albumin; Col, collagen; OA, osteoarthritis; PBS, phosphate buffered saline; P-PRP, pure platelet-rich plasma; PRP, platelet-rich plasma; PRPr, PRP releasate.

### Equine arthritis

One key limitation of the current arthritis models is that the cartilage pathology is created artificially rather than from natural diseases, which may undermine the justification of the clinical application of PRP. In a report by Carmona and colleagues [83], seven horses suffering severe joint diseases (four with OA, three with osteochondrosis) were treated with a cycle of three intra-articular injections of PRP at 2-week intervals after other conservative methods or arthroscopic interventions failed. Two months after the last injection, the synovial effusion and the degree of lameness in all seven horses were significantly reduced (*P* < 0.05), and the trend of symptomatic relief continued during the 1 year follow-up. In this study, however, cartilage changes were not monitored.

## Effect of platelet-rich plasma on human cartilage injury

On the basis of the strength of evidence, current published reports of PRP treatment of degenerative cartilage diseases may be divided into the following four levels: level IV, case series; level III, retrospective comparative studies; level II, prospective comparative studies or lesser quality randomized control trials (RCTs); and level I, high-quality RCTs [84,85].

### Focal cartilage lesions

Only a few reports on PRP treatment of focal cartilage defects have been published, all of which are case reports with a small number of patients and short-term results (Table 5). Thus, only low levels of evidence are provided.

**Table 5:** Summary of clinical studies of platelet-rich plasma for treatment of focal cartilage defects

**Patient number (age/range)**	**Defect position**	**Lesion size or grade**	**Innervation**	**Follow-up (months)**	**Outcome**	**Reference**
1 (12 years)	Medial femoral condyle	>2 cm^2^ full-thickness avulsion	Reattachment of loose body and P-PRP injection	9	Complete reattachment and perfect continuity on MRI at 18 weeks; return to soccer training at 18 weeks and fully involved in competition at 9 months	[86]
5 (21–37 years)	Femoral condyle	3-12 cm^2^, full-thickness	Cultured autologous BMSC + platelet-rich fibrin glue	14.2	All patients symptoms improved; ICRS nearly normal in 2 patients; MRI showed complete defect fill in 3 patients	[87]
5 (24–45 years)	Patellar cartilage	1-3 cm^2^; ICRS grade III or IV	Col I/III scaffold with L-PRP gel	24	VAS pain scores were reduced and function improved, but intralesional osteophytes in 3 patients and irregular surface were found in all	[88]
20 (15–50 years)	Knee osteochondral lesions	ICRS grade III or IV	HA membrane + BM concentrate + P-PRP gel	29	IKDC improved from 32.9 to 90.4; KOOS from 47.1 to 93.3; Col II positive and Col I negative staining in entire biopsies in 2 patients	[89]
48 (15–50 years)	Talar osteochondral lesions	1.6-2.6 cm^2^; 3–5 mm deep	Collagen or HA membrane + BM concentrate + P-PRP gel	29	AOFAS improved from 64.4 to 91.4; 94% return to low-impact sports at 4.4 months; varying regeneration on MRI and histological exam	[90]
52 (25–65 years)	Femoral and tibial condyle	1.5-5 cm^2^; Outerbridge III or IV	PGA-HA scaffold immersed in P-PRP and BM stimulation	12	All KOOS subscores improved; nearly normal appearance in 10 during arthroscopy; hyaline-like cartilage formation in 5 biopsies	[91]

AOFAS, American Orthopaedic Foot and Ankle Society; BM, bone marrow; BMSC, bone marrow-derived mesenchymal stem cell; Col, collagen; HA, hyaluronic acid; ICRS, International Cartilage Repair Society; IKDC, International Knee Documentation Committee; KOOS, Knee injury and Osteoarthritis Outcome Score; L-PRP, leukocyte- and platelet-rich plasma; MRI, magnetic resonance imaging; P-PRP, pure platelet-rich plasma; PGA, polyglycolic acid; VAS, visual analogue scale.

One case report described a 12-year-old soccer player who was diagnosed with a large (>2 cm^2^), loose chondral body avulsed from the medial femoral condyle [86]. After the loose body was placed in its bed, CaCl_2_-activated P-PRP was injected to fill up any mismatch between the crater and the fragment during arthroscopic surgery. Given the extremely poor prognosis of the larger chondral avulsion, which did not extend into the vascularized subchondral bone, this treatment was considered very successful, as the patient returned to soccer competition without any symptoms by 38 weeks postoperatively. The authors attributed the success to the addition of PRP, which augmented the reattachment of the cartilage fragment.

As MSCs play a crucial role in cartilage tissue engineering, Haleem and colleagues [87] seeded expanded autologous BMSCs into PRP gel to fill full-thickness cartilage defects in femoral condyles. Five patients aged 21 to 37 years were included, with their defects ranging from 3 to 12 cm^2^. All patients’ symptoms improved over the follow-up period of 12 months. Average Lysholm and Revised Hospital for Special Surgery Knee scores showed statistically significant improvement (*P* < 0.05). Arthroscopic scores recommended by the International Cartilage Repair Society were nearly normal in two patients who consented to a second-look arthroscopy. MRI of three patients revealed complete defect fill and surface congruity with native cartilage.

Considering the potential risks of culturing BMSCs *in vitro*, a few authors have been inclined to adopt microfracture or bone marrow concentrate to introduce BMSCs into the defects. Dhollander and colleagues reported on five patients who were treated with microfracture and L-PRP gel filling up the patellar cartilage defects ranging from 1 to 3 cm^2^[88]. The defects were sealed with Col I/III membranes. Symptoms and knee function of all five patients improved markedly after operation. However, such favorable results were not reflected by the MRI data, which showed subchondral lamina and bone changes in all five cases, and intralesional osteophytes in three at 2 year follow-up. In another case series of 20 patients, a composite of HA membrane, bone marrow concentrate and P-PRP gel was implanted into the osteochondral defects in the femoral condyle [89]. During the 29-month follow-up, the International Knee Documentation Committee (IKDC) scores improved from 32.9 to 90.4 (*P* < 0.0005), and the Knee injury and Osteoarthritis Outcome Scores (KOOS) improved from 47.1 to 93.3 (*P* < 0.0005). In two patients, who consented to a biopsy at 12 months postoperatively, cells were found to be homogeneously distributed, and were stained positively for Col II but negatively for Col I throughout the entire thickness of the biopsies, indicating the high quality of the regenerated cartilage. Another report confirmed the effectiveness of such a one-step repair of talar cartilage defects in 48 cases [90]. After implantation, the mean American Orthopaedic Foot and Ankle Society (AOFAS) scores improved steadily, from a pre-operative value of 64.4 to 83.3 at 6 months, 88.9 at 12 months, and 91.4 at 24 months. Among the 48 patients, 45 (94%) could participate in low-impact sports at a mean of 4.4 months, and 37 (77%) could participate in high-impact sports at a mean of 11.3 months.

Given the poor mechanical properties of the PRP gel, Siclari and colleagues [91] adopted polyglycolic acid-HA scaffolds immersed in autologous P-PRP to fill knee cartilage defects. At 9 months, all KOOS subscores improved in 52 patients, including pain, symptoms, activities of daily living, sports and recreation and quality of life subscales (*P* < 0.001). Histological evaluation of five patients showed a homogeneous hyaline-like cartilage repair tissue.

No complications related to PRP were noted in any of the studies described above during their follow-up.

### Degenerative joint diseases

#### Level IV case series

Among the case reports illustrated in Table 6[92-99], Wang-Saegusa and colleagues [99] reported the largest case series of knee OA treated with P-PRP intra-articular injections. A total of 808 patients were treated and 261 were evaluated after strictly applying the inclusion and exclusion criteria. At the end of 6 months, pain was significantly reduced while knee function and quality of life were improved, without occurrence of adverse events. The authors concluded that P-PRP injections had local, effective and temporal effects reducing pain and restoring function. However, the lack of a placebo group or conservatively managed control undermined the conclusions of these studies.

**Table 6:** Summary of clinical studies of platelet-rich plasma for treatment of degenerative cartilage lesions

**Level of evidence**^**a**^	**Patient number (age/range)**	**Intervention**	**Follow-up**	**Outcome**	**Adverse effects**	**Reference**
Level IV	14 (18–87 years)	3 L-PRP injections every 4 weeks	12 m	Significant and linear improvement in KOOS. Pain reduced after movement and at rest	Modest pain persisting for days	[92]
Level IV	17 (30–70 years)	Single PRP injection	12 m	Pain decreased, whereas function improved. MRI showed no worsening in 12 of 15 knees	Unreported	[93]
Level IV	27 (18–81 years)	3 weekly L-PRP injections	6 m	Substantial pain reduction after 1st injection and further improved at 6 months. WOMAC improved	No	[94]
Level IV	40 (33–84 years)	3 weekly P-PRP injections	6 m	Pain and disability subscores were significantly reduced	Transient sensation of hip heaviness	[95]
Level IV	50 (32–60 years)	2 L-PRP injections every month	12 m	IKDC and KOOS improved; all returned to previous activities	Unreported	[96]
Level IV	91 (24–82 years)	3 injections of double-spun PRP activated by CaCl_2_ every 3 weeks	12 m, 24 m	Pain decreased and knee function improved, especially in younger patients at 12 months. The improvements decreased at 24 months, but still better than the basal evaluation	Mild pain persisting for days	[97,98]
Level IV	261 (mean 48 years)	3 injections of CaCl_2_-activated P-PRP every 2 weeks	6 m	Significant differences in VAS, SF-36, WOMAC and Lequesne index	No	[99]
Level III	30 (36–76 years)	3 injections of double-spun PRP inactivated PRP or HA every 3 weeks	6 m	Both improved in IKDC, WOMAC and Lequesne index, but PRP exhibited better scores	Pain, swelling, but resolved in days	[100]
Level III	60 (61 years in HA, 64 years in PRP)	3 weekly injections of CaCl_2_-activated P-PRP or HA	5 w	33.4% patients in PRP group and 10% in HA achieved at least 40% pain reduction. Disability reduced more in PRP group than HA	Mild self-limiting pain and effusion in both groups	[101]
Level II	120 (19–77 years)	3 weekly L-PRP or HA injections	6 m	Better results in WOMAC and NRS in PRP than HA	Temporary mild worsening of pain	[102]
Level II	150 (26–81 years)	3 injections double-spun PRP or HA every 2 weeks	6 m	Higher IKDC but lower VAS pain scores than HA, especially in younger patients	No	[103]
Level II	32 (18–60 years)	3 injections of CaCl_2_-activated P-PRP or HA every 2 weeks	7 m	Higher AOFAS but lower VAS pain scores than HA	Mild pain, but self-resolved	[104]
Level I	78 (33–80 years)	Single or twice leukocyte-filtered PRP injection, or single saline injection	6 m	WOMAC improved after PRP injection, whereas worsened after saline infiltration	Self-resolved nausea and dizziness	[105]
Level I	120 (31–90 years)	4 weekly injections of inactivated P-PRP or HA	6 m	Significantly better clinical outcome and lower WOMAC scores than HA	None observed	[106]
Level I	176 (41–74 years)	3 weekly injections of CaCl_2_-activated P-PRP or HA	6 m	14.1% more patients reduced pain at least 50% in PRP group, with a significant difference	Mild, evenly in 2 groups	[107]
Level I	96 (50–84 years)	3 injections of CaCl_2_-activated P-PRP every 2 weeks, or single HA injection	48 w	Significantly more efficient in reducing pain, stiffness and improving physical function than HA	Mild, evenly in 2 groups	[108]
Level I	109 (18–80 years)	3 weekly injections of double-spun PRP releasate after freezing and thawing or HA	12 m	No significant difference in all scores. Only a trend favoring PRP in patients with early OA	Mild pain and effusion	[109]

^a^According to [84,85]. AOFAS, American Orthopaedic Foot and Ankle Society; HA, hyaluronic acid; IKDC, International Knee Documentation Committee; KOOS, Knee injury and Osteoarthritis Outcome Score; L-PRP, leukocyte- and platelet-rich plasma; m, months; MRI, magnetic resonance imaging; NRS, Numeric Rating Scale; P-PRP, pure platelet-rich plasma; PRP, platelet-rich plasma; SF, short form; VAS, visual analogue scale; w, weeks; WOMAC, Western Ontario and McMaster Universities Osteoarthritis Index.

#### Level III retrospective comparative studies

In a retrospective cohort of 30 patients sustaining chronic knee pain, the efficacy of PRP injections was compared with the more common, well-recognized HA treatment [100]. Patients received three intra-articular injections of inactivated double-spun PRP or HA every 3 weeks and were followed for 6 months after the final injections. Both groups showed significant improvement in IKDC, Western Ontario and McMaster Universities Osteoarthritis Index (WOMAC) and Lequesne Index, but the PRP-treated patients exhibited better results at 6 months than the HA-treated patients. Sánchez and colleagues [101] reported a similar cohort study to compare the therapeutic effect of P-PRP and HA for treatment of knee OA. Each group included 30 patients matched according to their age, sex, body mass index and OA severity on radiography. Results were only considered successful when a reduction of at least 40% from baseline in WOMAC pain scores occurred. In the PRP group, the success rate reached 33.4% at 5 weeks compared to only 10% in the HA group (*P* = 0.004). In addition, with respect to HA treatment, P-PRP injections significantly reduced the physical function subscale and the overall WOMAC (*P* = 0.043, *P* = 0.010, respectively) in favor of PRP treatment.

#### Level II prospective comparative studies or lesser quality randomized controlled trials

Two recent prospective cohort studies further confirmed the superiority of PRP over HA injections for treatment of knee OA [102,103]. Spaková and colleagues [102] reported 120 knee OA patients who were randomly treated with three injections of L-PRP or HA, one per week. At 3 and 6 months follow-up, better results in WOMAC and Numeric Rating Scale were obtained in the L-PRP group (*P* < 0.01). The other study included 150 patients suffering knee cartilage degenerative lesions, who were divided evenly into three groups [103]. These three homogeneous groups received three intra-articular injections of double-spun PRP, low-molecular-weight HA or high-molecular-weight HA. At 6 months follow-up, the best results in terms of IKDC, visual analogue scale and patient satisfaction were achieved in the PRP group (*P* < 0.005), in particular for the younger patients affected by cartilage lesions or early OA.

In an RCT comparing P-PRP and HA treatment in a total of 32 patients suffering talar osteochondral lesions [104], the mean AOFAS score was significantly improved from 68 and 66 before injection to 92 and 78 after 28 weeks in P-PRP and HA groups (*P* < 0.0001), respectively, favoring P-PRP treatment (*P* < 0.05). Better results for the visual analogue scale and other subjective function scores were also noted in the P-PRP group (*P* < 0.01).

#### Level I high-quality randomized controlled trials

A recent RCT compared PRP with placebo for the treatment of knee OA [105]. Seventy-eight patients (156 knees) were randomly divided into three groups. Group A received a single injection of P-PRP, group B received two injections of P-PRP 3 weeks apart, and group C received a single saline infiltration. After 6 months, all the subscores in WOMAC improved in groups A and B, but worsened in group C. These results support the short-term effectiveness of P-PRP injections over placebo for relieving pain and improving knee function.

The efficacy of PRP in the treatment of OA was also compared with HA administration. Cerza and colleagues [106] reported on 120 gonarthrosis patients undergoing 4 randomized intra-articular injections of P-PRP or HA. Patients in two groups were matched in terms of age, gender, severity of knee arthrosis and pre-treatment WOMAC scores. All patients were WOMAC evaluated before the infiltration and at 4, 12 and 24 weeks after the first injection. While post-treatment WOMAC scores in both groups significantly improved compared to before the infiltration, the improvement was more significant in the P-PRP-treated group than the HA group at each time point. In addition, the trend continued during the 24-week follow-up in the P-PRP group, but began to attenuate at 4 weeks in the HA group. These results indicated that P-PRP had a stronger and longer effect on the attenuation of OA with respect to HA treatment. In a multicenter, double-blind RCT, a total of 176 patients with symptomatic knee OA were randomly assigned to receive P-PRP or HA infiltrations [107]. The groups were well balanced for age, gender, body mass index, percentage of patients with primary arthritis, daily consumption of analgesics, radiographic grade, and WOMAC and Lequesne scores. The primary outcome measure was a 50% decrease in knee pain from baseline to week 24; according to this, the rate of response was 14.1% higher in P-PRP-treated patients compared to the HA-treated group. Regarding the secondary outcome measures assessing pain, stiffness and physical function, PRP also yielded better results than HA, albeit not reaching significance. Another recent RCT confirmed the superiority of P-PRP over HA in the alleviation of knee pain and stiffness and the improvement of physical function at both 24 and 48 weeks [108].

On the other hand, a single-center, double blind RCT including 109 matched patients demonstrated that PRP treatment did not lead to statistically significant differences in all scores evaluated with respect to HA injections at 12-month follow-up [109]. Further analysis showed a tendency favoring PRP in patients with less degenerated joints at 6 months and 12 months, although no significant difference was reached (*P* = 0.08 and *P* = 0.07, respectively). However, unlike the aforementioned RCTs, which used fresh P-PRP, this trial prepared PRP manually by double-spinning followed by freezing and thawing. Although the accurate concentration of leukocytes was unreported, it was estimated that this preparation concentrated leukocytes together with platelets and the final product was likely to contain much higher levels of pro-inflammatory signaling cytokines than P-PRP [46,110].

The unfavorable effect of concentrated leukocytes in PRP is also reflected in post-injection reactions. After intra-articular P-PRP injections, undetectable or only mild, self-resolved adverse events were observed, comparable to that observed with HA administration [106-108], but the double-spun PRP induced a significantly higher rate of pain reaction than the HA treatment (*P* = 0.039) [109]. A RCT comparing single- and double-spun PRP confirmed that the latter produced more pain and swelling reaction than the former, in which leukocytes were less concentrated [111].

## Conclusion

Research findings derived from basic and preclinical studies and from clinical trials collectively suggest that PRP is a promising treatment for cartilage injuries and relieving symptoms owing to its three known biological properties. Firstly, PRP has an anabolic effect on chondrocytes, MSCs and synoviocytes with resultant increases in cell proliferation, cartilaginous ECM accumulation, and HA secretion. Secondly, PRP may act as a bioactive cell scaffold to fill defects and enhance cartilage regeneration. Thirdly, PRP has the potential to inhibit inflammation and alleviate OA symptoms with a clinically acceptable safety profile. Although the majority of published evidence has favored PRP over HA for treatment of OA, PRP therapy remains unpredictable owing to the significant heterogeneity among studies and the variability in PRP preparations. Future studies are critical to elucidate the functional relationship between specific components of PRP and major pathogenic mechanisms.

## Abbreviations

AOFAS: American Orthopaedic Foot and Ankle Society; BMSC: Bone marrow-derived mesenchymal stem cell; Col: Collagen; ECM: Extracellular matrix; FBS: Fetal bovine serum; HA: Hyaluronic acid; HGF: Hepatocyte growth factor; IGF: Insulin-like growth factor; IKDC: International Knee Documentation Committee; IL: Interleukin; IL-1ra: IL-1 receptor antagonist; KOOS: Knee injury and Osteoarthritis Outcome Score; L-PRF: Leukocyte- and platelet-rich fibrin; L-PRP: Leukocyte- and platelet-rich plasma; MMP: Matrix metalloproteinase; MRI: Magnetic resonance imaging; MSC: Mesenchymal stem cell; NF-κB: Nuclear factor kappa B; OA: Osteoarthritis; PDGF: Platelet-derived growth factor; PLGA: Poly (lactic-co-glycolic acid); P-PRP: Pure platelet-rich plasma; PBS: Phosphate buffered saline; PPP: Platelet-poor plasma; PRF: Platelet-rich fibrin; PRP: Platelet-rich plasma; PRPr: PRP releasate; RCT: Randomized control trial; sTNF-R: Soluble TNF receptor; TNF: Tumor necrosis factor; VEGF: Vascular endothelial growth factor; WOMAC: Western Ontario and McMaster Universities Osteoarthritis Index.

## Competing interests

The authors declare that they have no competing interests.
